# Modeling Approach to Optimizing Dose Regimen of Vancomycin for Chinese Pediatric Patients with Gram-Positive Bacterial Infections

**DOI:** 10.3389/fphar.2021.648668

**Published:** 2021-04-15

**Authors:** Kai Shen, Yaxin Fan, Minjie Yang, Yuancheng Chen, Jinhao Tao, Guoping Lu, Hong Zhang, Qiwei Huang, Jing Zhang

**Affiliations:** ^1^ Institute of Antibiotics, Huashan Hospital, Fudan University, National Clinical Research Center for Aging and Medicine, Huashan Hospital, Fudan University, Key Laboratory of Clinical Pharmacology of Antibiotics, National Health and Family Planning Commission of People’s Republic of China, Shanghai, China; ^2^ Department of Critical Care Medicine, Children’s Hospital of Fudan University, Shanghai, China; ^3^ Department of Clinical Laboratory, Children’s Hospital of Shanghai Jiaotong University, Shanghai, China; ^4^ Department of Neonatology, Children’s Hospital of Shanghai Jiaotong University, Shanghai, China

**Keywords:** vancomycin, pediatric, pharmacokinetics/pharmacodynamics, population pharmacokinetics, external validation

## Abstract

The aim of this study was to establish the population pharmacokinetics (PK) model of Vancomycin for Chinese pediatric patients which can extrapolate to whole age periods by bridging the published adult population PK model and the established pediatric population PK model. The final consolidated population PK model was used to explore the correlation of pharmacokinetics/pharmacodynamics (PK/PD) indices and efficacy of vancomycin and to provide evidence for the optimized regimen of vancomycin in Chinese pediatric patients with Gram-positive bacterial infection. 108 pediatric patients with Gram-positive infections from 2 pediatric hospitals in China in the first period of the prospective multi-center vancomycin clinical observational study were enrolled to establish the population PK model. A one-compartment population PK model was established and validated. The correlation between vancomycin PK/PD indices [trough concentration (C_min_), peak concentration (C_max_), 0–24 h area under the curve (AUC_0–24_) and the area under the curve to minimum inhibitory concentration ratio (AUC_0–24_/MIC)] and the overall clinical outcomes (clinical efficacy and microbiological efficacy) in Chinese pediatric patients were evaluated. There is no significant correlation between PK/PD indices and clinical efficacy or microbiological efficacy. Considering the high clinical effective rate (>90%) and median AUC_0–24_/MIC values of 200–300, Chinese pediatric patients with Gram-positive bacterial infection may be suitable for lower AUC_0–24_/MIC target value compared to the target value of 400–600 recommended by IDSA guideline. Different optimal dose regimen of vancomycin for Chinese pediatric patients should be considered. Further evaluation in more prospective studies will be needed.

## Introduction

Since the 1980s, vancomycin has been the first-line treatment of methicillin-resistant *Staphylococcus aureus* (MRSA) infection. However, vancomycin has the characteristics of narrow therapeutic window, high inter-individual pharmacokinetic (PK) variability, and potential nephrotoxicity and ototoxicity. Therapeutic drug monitoring (TDM) has been routinely used in clinical practice to optimize efficacy and safety of vancomycin. In March 2020, the American Society of Health-System Pharmacists (ASHP), the Infectious Diseases Society of America (IDSA), the Pediatric Infectious Diseases Society (PIDS), and the Society of Infectious Diseases Pharmacists (SIDP) published the revised consensus guideline for therapeutic monitoring of vancomycin for MRSA infection (Rybak et al., 2020). In this consensus guideline, predictive target value of the area under the curve to minimum inhibitory concentration ratio (AUC_0–24_/MIC) based on population pharmacokinetic analysis combined with Bayesian approaches was recommended for therapeutic drug monitoring, and trough concentration monitoring alone is no longer recommended. The target value of AUC_0–24_/MIC recommended by the consensus guideline is 400–600, for both adults and pediatric patients.

However, the recommended target value of AUC_0–24_/MIC and trough concentration has always been controversial due to insufficient evidence for efficacy and safety (Lodise et al., 2009; Rybak et al., 2009; Kullar et al., 2012; Gawronski et al., 2013; Holmes et at., 2013). The investigations of vancomycin pharmacokinetics/pharmacodynamics (PK/PD) in Chinese patients are mostly based on retrospective observational clinical studies, which provided limited clinical efficacy and safety evidence. There are even fewer vancomycin PK/PD studies conducted in Chinese pediatric patients. Most of these studies only established population PK model in newborn patients and did not evaluate PK/PD combined with clinical outcome or microbial efficacy. There is an urgent need to conduct a prospective large sample size clinical research to provide evidence for optimizing dose regimen of vancomycin for adults and pediatric patients in China.

Our research is to establish population PK model utilizing the clinical data of pediatric patients from a prospective, pathogen diagnosis–based, multicenter, observational study (Liang et al., 2018; Shen et al., 2018), assess the clinical and microbiological efficacy of vancomycin, and recommend optimized dose regimen of vancomycin in Chinese pediatric patients.

## Method

### Study Design

The research data were from a prospective, multicenter, randomized, open label clinical observational study (Period I) of vancomycin for the treatment of patients with Gram-positive bacterial infection. All the patients had clinical and microbiological evidence (clinical symptoms, signs, laboratory tests, and microbiology culture) for the diagnosis. Patients from 13 hospitals in China including 2 pediatric hospitals with Gram-positive infections who received vancomycin therapy ≥5 days and who were under therapeutic drug monitoring (TDM) were enrolled in this study. Patients received any other agents that are effective against Gram-positive bacterial infection for ≥24 h within 72 h of receiving vancomycin therapy and patients who were considered to have Gram-positive bacteria colonization were excluded.

The study was approved by the medical ethics committee of each study center and was performed in accordance with the ethical standard established by the 1964 Declaration of Helsinki and its later amendments. Written informed consent was obtained from all enrolled patients or their legally authorized representatives. The study was registered with the Chinese Clinical Trial Registry (www.chictr.org.cn, number ChiCTR-OPC-16007920).

### Laboratory Test

The TDM concentration data of therapeutic drugs in adults and pediatric patients with Gram-positive bacterial infection were collected. Serum samples were collected within 0.5 h before the fifth dose of vancomycin, and at any point from 0.5–1 h after the fifth dosing of vancomycin. The bioanalysis method for vancomycin TDM was a fluorescence polarization immunoassay (FPIA) or a chemiluminescence immunoassay (CMIA) with a calibration range of 3.00∼100 mg/L. The minimum inhibitory concentrations (MIC) of vancomycin was verified by the agar dilution method in a CHINET microbiology laboratory. Clinical Laboratory Standards Institute (CLSI) protocols M07-A9 and M100-S24 were performed as MIC testing standards. MIC_50_ and MIC_90_ values were defined as the lowest concentration of vancomycin at which 50 and 90% of the isolates were inhibited, respectively.

### Clinical Outcomes Evaluation

Both clinical efficacy (improvement of symptoms and signs of infection) and microbiological response (bacteria eradication) were evaluated in all the eligible patients. The clinical efficacy was evaluated centrally by the investigator with double check. The eradication of bacteria was defined as the inability to culture the original pathogen at the primary infection site and the absence of the need for anti-gram-positive bacterial antibiotic within 7 days after the end of the vancomycin treatment.

### Population PK Modeling and Simulation

The population PK model of vancomycin for adults and pediatric patients were established respectively to investigate the difference of covariates using NONMEM 7.3.0 (ICON Development Solutions, Ellicott City, MD). The population pharmacokinetics model was composed of a structural model and random effect models using the first-order conditional estimation method (FOCE) with interaction. Demographic variables (e.g., gender, age, weight, height, body mass index [BMI]), renal function descriptors (serum creatinine, eGFR, creatinine clearance rate [CLCr], and albumin, etc.), and disease conditions (e.g., surgery or injury, chronic kidney disease, diabetes, cancer) were tested as potential covariates. Covariates were evaluated using the stepwise forward-selection method and backward elimination. The population PK model for adults and pediatric patients were evaluated respectively and the model parameters were compared. In order to unify the population PK model for adults and pediatric patients, the datasets were merged and the covariates were consolidated and reevaluated. By introducing body weight as scaling factor, an unified final population PK model for the pooled data of adults and pediatric patients was established and validated. External validation was performed using part of the data from Period II of the vancomycin observational study. The established population PK model was used to simulate individual PK parameters (C_min_, C_max_, AUC_0–24_) by Bayesian feedback method for the further PK/PD evaluation.

Utilizing Monte Carlo simulation, the PK/PD index of AUC_0–24_/MIC of vancomycin for Chinese pediatric patients at different ages under different administration scheme were simulated. The optimal dose regimens were recommended for the treatment of Gram-positive bacterial infection in Chinese pediatric patients at different ages.

### Statistical Analysis

Multivariate logistic regression analysis was carried out with SAS 9.4 (SAS Institute, Inc: Cary, NC) to evaluate the correlation between the PK/PD indices (C_min_, C_max_, AUC_0–24_ and AUC_0–24_/MIC) of vancomycin and the overall efficacy (clinical efficacy and microbial efficacy). The χ2 test was used to compare categorical variables. *p* < 0.05 was considered significant.

## Results

In total, 108 pediatric patients with Gram-positive infections from 2 pediatric hospitals in China from the prospective multi-center vancomycin clinical observational study were enrolled to establish the population PK model, and the dataset for model development used 251 observations, including trough and peak concentrations at steady state. The demographics and baseline clinical characteristics of patients were shown in Table 1. In our study, most of the pediatric patients were neonates and infants (<3 years old, *n* = 90). There were 16 patients from 3 years old to 12 years old, and only 2 cases were older than 12 years old.

**TABLE 1:** Demographic and baseline characteristics of the pediatric patients included in the population PK analysis (*n* = 108).

Variables	Mean ± SD	Median (range)
Age (years)	1.47 ± 2.63	0.456 (0.0164–13.0)
Body weight (kg)	8.47 ± 9.22	5.40 (0.900–55.0)
Height (m)	0.688 ± 0.278	0.600 (0.300–1.70)
BMI (kg/m^2^)	14.1 ± 4.66	14.7 (3.60–34.3)
BSA (kgm^2^)	0.388 ± 0.287	0.289 (0.132–1.65)
S_CR_(umol/L)	25.6 ± 14.3	20.0 (9.00–83.0)
TBIL (umol/L)	29.8 ± 43.4	9.05 (2.00–218)
ALT (U/L)	21.9 ± 23.9	13.0 (1.00–129)
AST (U/L)	34.1 ± 43.3	24.0 (7.00–418)
ALB (g/L)	35.1 ± 6.19	36.0 (2.90–47.0)
WBC (e^9^/L)	6.56 ± 2.93	6.00 (2.41–11.4)
ENC (%)	47.8 ± 14.3	48.3 (1.20–93.3)

BMI: body mass index; BSA: body surface area; S_CR_: serum creatinine; TBIL: total bilirubin; ALT: alanine aminotransferase; AST: aspartate aminotransferase; ALB: albumin; WBC: white blood cell; ENC: eosinophils cell.

To minimize the distribution bias of different age period, extrapolation from adult population PK model to pediatric population PK model was investigated. In our previous research, a population pharmacokinetic model of vancomycin in Chinese adult patients was established using 380 adult patients from the prospective multi-center vancomycin clinical observational study (Shen et al., 2018). The population PK datasets of adults and pediatric patients were combined and evaluated the covariates related to age and physiology (such as CLCr, weight, body surface area, etc.) and finally established a unified population PK model for adults and pediatric patients. By comparing the unified population PK models and original model, the weight was introduced as the covariate on the basis of the population PK model of adults. The final population model and typical pharmacokinetic parameters are as follows:

$$\mathrm{CL}\left(L/h\right)=3.83\times {\left(\frac{\mathrm{CLCr}}{90.28}\right)}^{0.516}\times {\left(\frac{\mathrm{WT}}{58.25}\right)}^{0.646}\times e^{\eta_{CL}}$$ (1)

$$V\left(L\right)=44.7\times {\left(\frac{\mathrm{AGE}}{55}\right)}^{0.33}\times {\left(\frac{\mathrm{WT}}{58.25}\right)}^{0.349}\times e^{\eta_{V}}$$ (2)

The parameter estimates of the final model and bootstrap confidence intervals were shown in Table 2. The results showed that the estimated values of the final model parameters and the 95% confidence interval are very similar to the bootstrap results of 1,000 times of simulation, indicating that the performance of the model is very stable. External validation was also performed using the extra 23 pediatric patients from the 2^nd^ period of the observational study and showed good consistency between the predicted individual concentration and the observed concentration.

**TABLE 2:** Parameter estimates and bootstrap of the final population PK model of vancomycin for adults and pediatric patients.

Parameters		Final model				Bootstrap	
		Estimate	RSE (%)	95% CI	Shrinkage (%)	Median	95% CI
*θ*1	CL (L/h)	3.83	2.3	3.655–4.005	–	3.82	3.64–4.00
*θ*2	V (L)	44.7	3	42.093–47.307	–	44.8	42.2–47.5
*θ*3	CLCr effect on CL	0.516	7.1	0.444–0.588	–	0.519	0.450–0.593
*θ*4	Body weight effect on CL	0.646	4.1	0.594–0.698	–	0.644	0.590–0.696
*θ*5	Age effect on V	0.33	15.7	0.229–0.431	–	0.331	0.223–0.435
*θ*6	Body weight effect on V	0.349	30.4	0.141–0.557	–	0.347	0.129–0.567
*ω*1	CL IIV	0.204	9.8	–	9.6	0.201	0.164–0.242
*ω*2	V IIV	0.0427	41	–	56.2	0.0416	0.00598–0.0914
*σ*1	Residual error	0.0749	99.7	–	19.9	0.0746	0.0586–0.0893

RSE: relative standard error; CL: clearance; V: volume of distribution; BSA: body surface area; eGFR: estimated glomerular filtration rate; ALB: albumin; IIV: inter-individual variability.

**FIGURE 1:**
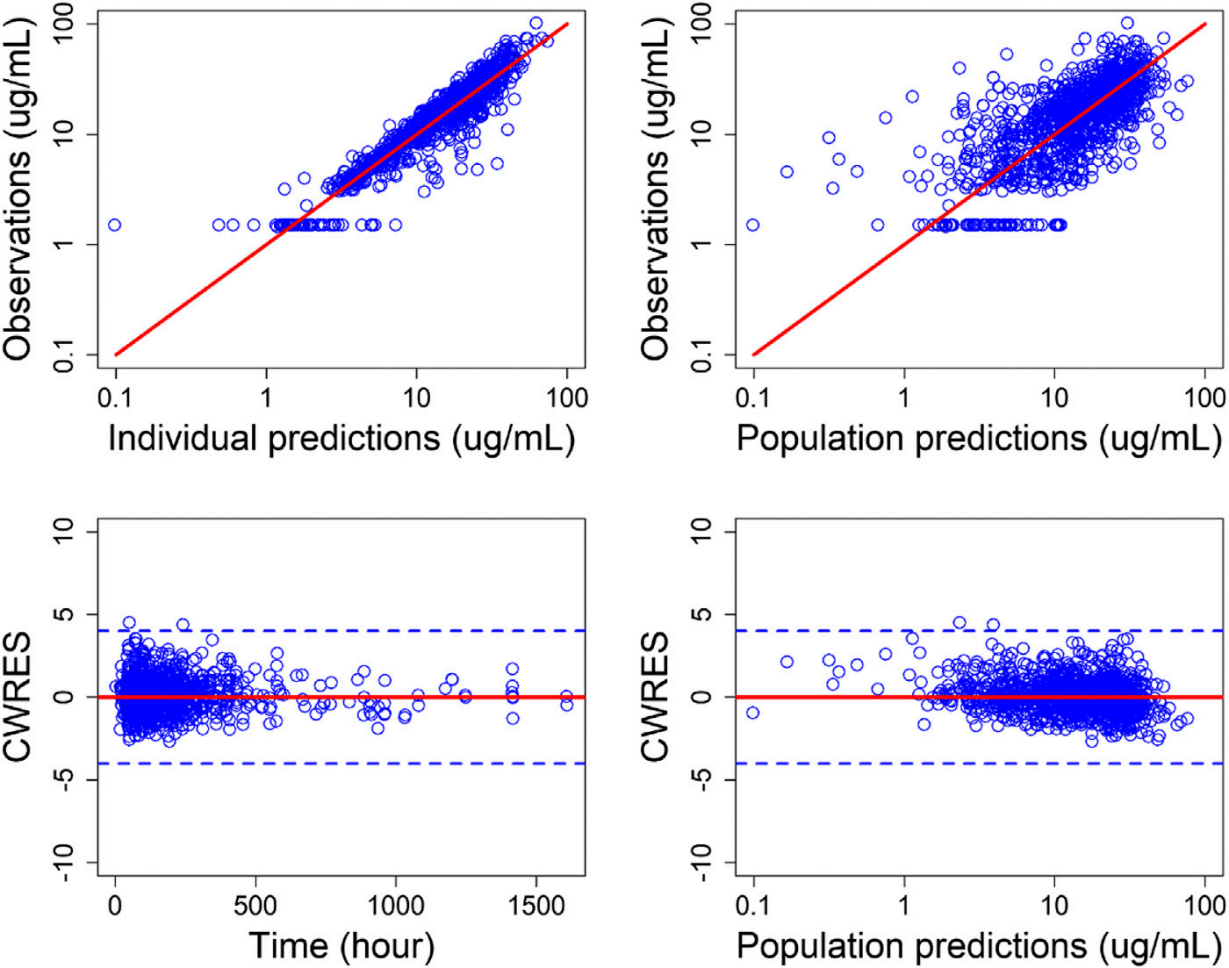
Goodness-of-fit plots for final population PK model. Top: Individual predicted (IPRED) serum concentrations vs. observed concentrations **(left)** and population predicted (PRED) serum concentrations vs. observed serum concentrations **(right)**. Bottom: conditional weighted residuals (CWRES) vs. time **(left)** and PRED **(right)**. Points are individual data. Red solid lines represent the unit diagonal **(top)** or line at zero **(bottom)**. Blue dashed lines represent |CWRES| of 4.

**FIGURE 2:**
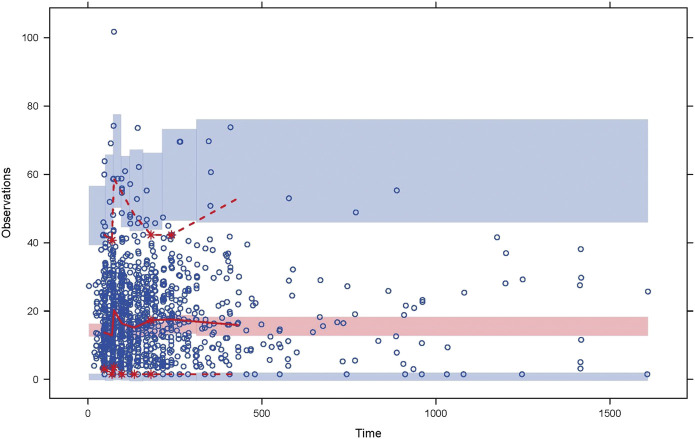
Visual predictive check for the final population pharmacokinetic model. The circles are the observations. The solid and dashed lines represent the median, 2.5th, and 97.5th percentiles of the observations; the shaded pink and blue areas represent the 95% confidence interval of the median, 2.5th, and 97.5th percentiles predicted by the model.

The goodness-of-fit plots for final population PK model were shown in Figure 1. A good agreement between the predicted concentrations and the observed concentrations was observed. The visual predictive check (VPC) for the final population PK model (Figure 2), showed that the model can predict the central tendency of the observed PK concentrations well. In general, the final population PK model has good prediction performance and can be used for further PK/PD evaluation.

Among the enrolled pediatric patients, the total cases with evaluable clinical efficacy was 108, and the number of cases with evaluable microbiological efficacy was 102. MRSA isolates from the pediatric patients had a vancomycin MIC_50_ = 1 mg/L and MIC_90_ = 1 mg/L. Both methicillin resistant coagulase-negative staphylococci (MRCNS) and *Enterococcus* species had vancomycin MIC_50_ = 1 mg/L and MIC_90_ = 2 mg/L.

The correlation between the PK/PD index of vancomycin (C_min_, C_max_, AUC_0–24_ and AUC_0–24_/MIC) and the overall clinical outcomes (clinical efficacy and microbial efficacy) were analyzed by multivariable logistic regression analysis (Tables 3 and 4). There were no significant correlation between the PK/PD indices of vancomycin and clinical/microbiological efficacy (*P* > 0.05).

**TABLE 3:** Multivariable logistic regression analyses on clinical/microbiological efficacy of vancomycin therapy.

Variable	Bacterial eradication		Clinical efficacy	
	Wald χ2	*P* Value	Wald χ2	*P* Value
Infection sites	9.9627	0.1907	0.4141	0.9997
Bacterial classification	0.8128	0.9992	7.5118	0.4825
C_min, ss_	1.0589	0.3035	0.1499	0.6987
C_max, ss_	0.4599	0.4977	1.9485	0.1628
AUC_0–24_	0.0507	0.8218	1.7681	0.1836
AUC_0–24_/MIC	1.1411	0.2854	NA	NA

C_min, ss_: trough concentration at steady state; C_max, ss_: peak concentration at steady state; AUC_0–24_: 0–24 h area under the curve; AUC_0–24_/MIC: the area under the curve to minimum inhibitory concentration ratio.

**TABLE 4:** Comparison of pharmacokinetic parameters and clinical efficacy (improvement of clinical signs and symptoms) by the most common infected sites.

Infected site	PK parameters	Improved	Not improved	*P* value
Overall	Responding, n (%)	100 (92.6)	8 (7.4)	
	C_min_,_ss_ (mg/L),Median (IQR)	1.50 (1.50, 5.75)	1.50 (1.50, 4.92)	0.8206
	C_min_,_ss_ (mg/L),Median (IQR)	21.50 (16.89, 27.12)	26.02 (22.01, 28.67)	0.1146
	AUC_0–24_, (hr*mg/L), median (IQR)	217.5 (172.87, 286.34)	212.6 (204.20, 314.50)	0.4082
Bloodstream	Responding, *n* (%)	49 (96.1)	2 (3.9)	
	C_min_,_ss_ (mg/L),Median (IQR)	1.50 (1.50, 5.95)	1.50	0.2650
	C_max,ss_ (mg/L),Median (IQR)	23.32 (17.20, 26.57)	26.02	0.3693
	AUC_0–24_, (hr*mg/L), median (IQR)	215.9 (172.33, 305.85)	225.0	>0.9999
Pulmonary	Responding, *n* (%)	27 (90.0)	3 (10.0)	
	C_min_,_ss_ (mg/L),Median (IQR)	3.02 (1.50, 6.17)	3.32	0.8553
	C_max,ss_ (mg/L),Median (IQR)	20.24 (16.24, 28.07)	22.60	0.6783
	AUC_0–24_, (hr*mg/L), median (IQR)	219.2 (183.44, 266.38)	213.1	0.5802
Urinary tract	Responding, *n* (%)	16 (94.1)	1 (5.9)	
	C_min_,_ss_ (mg/L),Median (IQR)	1.50 (1.50, 5.99)	1.50	0.4930
	C_max,ss_ (mg/L),Median (IQR)	22.08 (15.80, 25.63)	29.18	0.1846
	AUC_0–24_, (hr*mg/L), median (IQR)	196.2 (145.03, 287.92)	212.1	0.9187
Central nerve system	Responding, *n* (%)	10 (90.9)	1 (9.1)	
	C_min_,_ss_ (mg/L),Median (IQR)	4.77 (1.50, 10.58)	1.50	0.3315
	C_max,ss_ (mg/L),Median (IQR)	28.11 (24.75, 30.66)	21.41	0.4292
	AUC_0–24_, (hr*mg/L), median (IQR)	303.3 (204.97, 386.18)	200.1	0.4292
Endocarditis	Responding, *n* (%)	6 (100.0)	0 (0.0)	
	C_min_,_ss_ (mg/L),Median (IQR)	1.50 (1.50, 3.63)		
	C_max,ss_ (mg/L),Median (IQR)	21.50 (17.20, 23.70)		
	AUC_0–24_, (hr*mg/L), median (IQR)	213.4 (195.3, 239.1)		

C_min, ss_: trough concentration at steady state; C_max, ss_: peak concentration at steady state; AUC_0–24_: 0–24 h area under the curve; AUC_0–24_/MIC: the area under the curve to minimum inhibitory concentration ratio; IQR: interquartile range.

Data are presented as the median (IQR) or *n* (%); IQRs were not reported for *n* < 5.

Table 5 summarized bacterial response of vancomycin and AUC_0–24_/MIC by the most common infection sites and bacteria, which compared the difference (*P* value shows the statistical significance level) between the bacterial classifications. Although the results in Table 5 showed that the AUC_0–24_/MIC of different bacterial classification are statistically different (*p* < 0.05) in bloodstream, lung and urinary tract infections, there is no statistically significant difference in bacterial response between the different bacterial classifications. Therefore, the difference of AUC_0–24_/MIC between different bacterial classifications has no clinical significance.

**TABLE 5:** Summary of bacterial response of vancomycin and AUC_0–24_/MIC by the most common infection sites and bacteria.

Bacterial classification	*N*	Bacterial eradication		AUC_0–24_/MIC	
		*n* (%)	*P* value*	Median (IQR)	*P* value**
Bloodstream			1.0000		0.0039
SA	8	8 (100.0)		315.0 (215.5, 367.0)	
CoNS	32	31 (96.9)		187.0 (152.0, 250.5)	
*Enterococcus*	7	7 (100.0)		214.0 (184.0, 528.0)	
Other	4	4 (100.0)		553.5 (470.0, 746.0)	
Pulmonary			1.0000		0.0382
SA	24	23 (95.8)		220.5 (201.0, 301.0)	
CoNS	1	1 (100.0)		186.0 (186.0, 186.0)	
*Enterococcus*	1	1 (100.0)		69.0 (69.0, 69.0)	
Other	3	3 (100.0)		833.0 (660.0, 1060.0)	
Urinary tract			NA		0.0322
SA	2	2 (100.0)		138.0 (137.0, 139.0)	
CoNS	2	2 (100.0)		261.5 (233.0, 290.0)	
*Enterococcus*	11	11 (100.0)		179.0 (145.0, 286.0)	
Central Nerve System			0.0909		0.2012
SA	1	0 (0.0)		200.0 (200.0, 200.0)	
CoNS	2	2 (100.0)		276.0 (166.0, 386.0)	
*Enterococcus*	2	2 (100.0)		512.0 (178.0, 846.0)	
Other	6	6 (100.0)		1082.5 (820.0, 1313.0)	
Endocarditis			NA		0.7633
SA	1	1 (100.0)		195.0 (195.0, 195.0)	
CoNS	1	1 (100.0)		240.0 (240.0, 240.0)	
*Enterococcus*	1	1 (100.0)		196.0 (196.0, 196.0)	
Other	3	3 (100.0)		462.0 (188.0, 478.0)	

* Fishers’ Exact test.

** Maximum likelihood ratio test.

SA: *Staphylococcus aureus*; CoNS: *Coagulase-Negative Staphylococcus*; AUC_0–24_/MIC: 0–24 h area under the curve to MIC ratio; IQR: interquartile range; NA: not applicable.

**TABLE 6:** Population PK model predicted probability of target attainment (PTA) of vancomycin in pediatric patients at different ages under different dosing regimens and different target values.

AUC_0–24_/MIC target value	Age period	Dose regimen	MIC (mg/L)				
		(q6h, q8h or q12h) (mg/kg/day)	0.125	0.25	0.5	1	2
200	0–3m	40	**100**	**100**	**98.6**	66.7	17.4
		50	**100**	**100**	**100**	81.9	31.9
		60	**100**	**100**	**100**	89.9	44.2
		70	**100**	**100**	**100**	**94.2**	57.2
		80	**100**	**100**	**100**	**98.6**	66.7
	3m–12y	40	**100**	**100**	**100**	76.7	26.1
		50	**100**	**100**	**100**	88.9	43.3
		60	**100**	**100**	**100**	**96.1**	56.7
		70	**100**	**100**	**100**	**99.4**	65.6
		80	**100**	**100**	**100**	**100**	76.7
	12y -17y	40	**100**	**100**	**100**	**91.0**	46.5
		50	**100**	**100**	**100**	**99.0**	66.0
		60	**100**	**100**	**100**	**100**	79.5
		70	**100**	**100**	**100**	**100**	85.5
		80	**100**	**100**	**100**	**100**	**91.0**
250	0–3m	40	**100**	**100**	**92.0**	50.7	9.4
		50	**100**	**100**	**98.6**	66.7	17.4
		60	**100**	**100**	**100**	81.2	30.4
		70	**100**	**100**	**100**	87.7	39.1
		80	**100**	**100**	**100**	**92.0**	50.7
	3m–12y	40	**100**	**100**	**97.8**	61.1	16.7
		50	**100**	**100**	**100**	76.7	26.1
		60	**100**	**100**	**100**	86.7	38.9
		70	**100**	**100**	**100**	**92.2**	50.6
		80	**100**	**100**	**100**	**97.8**	61.1
	12y -17y	40	**100**	**100**	**100**	83.0	26.5
		50	**100**	**100**	**100**	**91.0**	46.5
		60	**100**	**100**	**100**	**97.0**	61.0
		70	**100**	**100**	**100**	**100**	75.0
		80	**100**	**100**	**100**	**100**	83.0
300	0–3m	40	**100**	**100**	84.1	35.5	3.6
		50	**100**	**100**	**93.5**	54.3	10.1
		60	**100**	**100**	**98.6**	66.7	17.4
		70	**100**	**100**	**100**	79.0	26.8
		80	**100**	**100**	**100**	84.1	35.5
	3m–12y	40	**100**	**100**	**90.6**	47.2	8.9
		50	**100**	**100**	**98.3**	62.8	18.3
		60	**100**	**100**	**100**	76.7	26.1
		70	**100**	**100**	**100**	85.0	36.1
		80	**100**	**100**	**100**	**90.6**	47.2
	12y–17y	40	**100**	**100**	**99.5**	70.5	17.5
		50	**100**	**100**	**100**	83.5	29.5
		60	**100**	**100**	**100**	**91.0**	46.5
		70	**100**	**100**	**100**	**96.5**	58.5
		80	**100**	**100**	**100**	**99.5**	70.5
400	0–3m	40	**100**	**98.6**	66.7	17.4	0.7
		50	**100**	**100**	81.9	31.9	2.9
		60	**100**	**100**	89.9	44.2	5.8
		70	**100**	**100**	**94.2**	57.2	10.9
		80	**100**	**100**	**98.6**	66.7	17.4
	3m–12y	40	**100**	**100**	76.7	26.1	3.3
		50	**100**	**100**	88.9	43.3	7.8
		60	**100**	**100**	**96.1**	56.7	14.4
		70	**100**	**100**	**99.4**	65.6	19.4
		80	**100**	**100**	**100**	76.7	26.1
	12y–17y	40	**100**	**100**	**91.0**	46.5	5.5
		50	**100**	**100**	**99.0**	66.0	14.0
		60	**100**	**100**	**100**	79.5	23.5
		70	**100**	**100**	**100**	85.5	34.0
		80	**100**	**100**	**100**	**91.0**	46.5

q6 h: every 6 h; q8h: every 8 h; q12 h: every 12 h. Bold text: target attainment above 90%.

The median value of AUC_0–24_/MIC was between 200 and 300, which did not reach the target value of 400, but the overall clinical effective rate was 92.6%. It suggested that the AUC_0–24_/MIC value of Chinese pediatric patients may not need to reach the target level (above 400) required by the guidelines. This observation is similar as the results of our previous research in adult patients (Shen et al., 2018).

Table 6 listed the results of the population PK model predicted probability of target attainment (PTA) of vancomycin in pediatric patients at different ages under different dosing regimens and different target values. It can be seen that lower daily dose in the >12-year-old age group can achieve the same target value compared with the <12-year-old age group.

Combined with the actual clinical efficiency, the optimal dose regimen for the treatment of in Chinese pediatric patients with Gram-positive infections is recommended to be 60–80 mg/kg/day every 6 or 8 h for <12-year-old and 50–60 mg/kg/day every 6 or 8 h for >12-year-old pediatric patients.

## Discussion

Vancomycin is mainly excreted through the kidneys. In the previous adult population PK model (Zhou et al., 2016; Shen et al., 2018), creatinine clearance (CLCr) was identified as a major covariate affecting the clearance (CL) of vancomycin. However, among the previous reported the pediatric vancomycin population PK models (Liu et al., 2017; Zane et al., 2017), only a small part of the models believe that creatinine clearance is a factor affecting vancomycin CL. The is mainly due to the children’s serum level of creatinine does not fully reflect the level of kidney function. In this study, the adult population PK model of vancomycin and the pediatric population PK model were firstly established and validated independently, and the covariate effects in adult and pediatric infection patients were investigated respectively. In the unified model of adults and pediatric patient, both CLCr and weight are considered as covariates. Zhou et al. (2016) studied the establishment of population PK model of renal clearance drugs, in which the vancomycin population PK model used CLCr and body weight as the main covariates to extrapolate from adult to children, which is the same as this study.

In this study, we successfully established the unified population PK model of vancomycin for adults and pediatric patients, and verified it as a tool model to extrapolate from adults to children, which meets the needs of different physiological mechanisms and therapeutic drug monitoring in clinical practice. In clinical practice, adult PK data is easier to obtain than children. Generally, the integrity and sufficiency of PK data of adult patients better than that of pediatric patients. At the same time, it can reduce the sampling bias caused by the uneven age distribution of pediatric PK dataset. The Bayesian feedback method can better predict the individual PK parameters of pediatric patients, and can also be used to extrapolate the PK/PD index of pediatric patients at different ages.

This study evaluated not only the clinical efficacy but also the microbiological efficacy, which was relatively rare in previous studies. In this study, univariate and multivariate logistic regression analysis was carried out on the correlation between (C_min_, C_max_, AUC_0–24_ and AUC_0–24_/MIC) with clinical efficacy (clinical efficacy + microbiological efficacy). In general, there was no significant correlation between vancomycin (C_min_, C_max_, AUC_0–24_ and AUC_0–24_/MIC) according to different infection sites and bacterial types. The clinical/microbiological effective in this study were very high (>90%), and only very few ineffective cases, which may be the reason of no significant correlation could be found between effectiveness and PK/PD indexes. Further evaluation will be needed based on accumulative data generated from the larger population in more prospective studies.

Based on the predicted AUC_0–24_/MIC target value and trough concentration of vancomycin in children with population PK model, combined with the actual clinical effective rate of Chinese children with infection, it is recommended that the optimal dosage regimen for the treatment of Chinese pediatric patients with Gram-positive infections is 60–80 mg/kg/day every 6 or 8 h (<12 years old), and 50–60 mg/kg/day every 6 or 8 h (>12 years old). The optimal dose regimen for Chinese pediatric patients younger than 12 years old is basically consistent with the recommended dose of IDSA, while the recommended dose for Chinese pediatric patients over 12 years old is slightly lower compared with the IDSA guideline. More prospective studies need to be performed to confirm these results.

## Conclusion

No significant correlations were identified between the PK/PD indices of vancomycin and clinical or microbiological efficacy in Chinese pediatric patients with Gram-positive infections in this prospective study. Based on our research, Chinese pediatric patients with infection may be suitable for lower AUC_0–24_/MIC target value compared to the IDSA guideline, and different optimal dose regimen of vancomycin for Chinese pediatric patients should be considered.

## Data Availability

The raw data supporting the conclusions of this article will be made available by the authors, without undue reservation.
